# Inside-out sustainability: The neglect of inner worlds

**DOI:** 10.1007/s13280-019-01187-w

**Published:** 2019-04-24

**Authors:** Christopher D. Ives, Rebecca Freeth, Joern Fischer

**Affiliations:** 1 School of Geography, University of Nottingham, University Park, Nottingham, NG7 2RD UK; 2 Faculty of Sustainability, Leuphana University Lueneburg, Scharnhorststr.1, 21335 Lueneburg, Germany

**Keywords:** Interiority, Leverage points, Religion, Spirituality, Sustainability transformation, Values

## Abstract

In the context of continuing ecosystem degradation and deepening socio-economic inequality, sustainability scientists must question the adequacy of current scholarship and practice. We argue that pre-occupation with external phenomena and collective social structures has led to the neglect of people’s ‘inner worlds’—their emotions, thoughts, identities and beliefs. These lie at the heart of actions for sustainability, and have powerful transformative capacity for system change. The condition of people’s inner worlds ought to also be considered a dimension of sustainability itself. Compassion, empathy and generosity, for example, are personal characteristics that mark individual expressions of sustainability. Sustainability science must take inner life more seriously by considering how language shapes and is shaped by paradigms about the world, prioritising enquiry into how spirituality, contemplation and sustainability transformation relate, and encouraging scholars and practitioners to intentionally cultivate their inner worlds to strengthen inner resources necessary for addressing sustainability challenges.

## Introduction

“I used to think the top environmental problems were biodiversity loss, ecosystem collapse and climate change. I thought with 30 years of good science we could address those problems. But I was wrong. The top environmental problems are selfishness, greed and apathy… And to deal with these we need a spiritual and cultural transformation - and we scientists don’t know how to do that.”

*-*James Gustave Speth

Sustainability science has come a long way in the last 20 years. Since Kates et al. (2001) published their pioneering essay, sustainability science has burgeoned as an integrative and applied discipline. Bringing together economics, social science, ecology and technology studies (Komiyama and Takeuchi 2006), the quest began to solve the most pressing practical and ethical challenges facing the planet and to address them via appropriate policies. Indeed, sustainability has moved from a buzzword to a mainstay concept in nearly all areas of society. However, despite the prominence of sustainability as a concept, planetary trajectories remain deeply unsustainable (e.g. WWF 2016).

Now that sustainability science is well established as a field of scholarship, it is timely to consider how it has progressed and where the field needs to go in the future. This article contends that despite substantial analytical advancement, sustainability scholarship has not catalysed the necessary change. The vast majority of sustainability science has focused on the external world of ecosystems, economic markets, social structures and governance dynamics. In doing so, a critical second dimension of reality has been neglected: the inner lives of individuals. We argue here that our inner worlds, such as our emotions, thoughts, identities and beliefs, lie at the root of sustainability challenges and are fundamental to the solutions to some of the world’s greatest challenges. Yet, apart from a few scattered examples (e.g. Wolf 2012; Horlings and Padt 2013), the inner life has evaded explicit analysis within mainstream sustainability science because it cannot be understood via traditional scientific tools, approaches and terminologies.

Some fields of knowledge have long recognised the importance of inner dimensions of human experience. Aristotle’s concept of *Phronesis* (or ‘practical wisdom’) is an important concept in classical philosophy. Practical wisdom has an inner source. One acquires an intuitive kind of knowledge, borne of experience, that enables action in uncertain or unprecedented situations (Harding 2009). Another foundational philosophical theory is David Hume’s theory of motivation (Hume 1975). Hume asserted that the motivation to perform some action is dependent on both an inner belief that the action is right, and the desire to perform it.

However, only more recently have environmental and sustainability scholars started to attend to inner worlds. A topic that has received considerable attention is the notion of value shift as integral to combating the environmental crisis. Martin et al. (2016, p. 6105) suggested that “we need fundamental shifts in values that ensure transition from a growth-centered society to one acknowledging biophysical limits and centered on human well-being and biodiversity conservation”. This is a call for change progressing from the *inside out* (see O’Brien 2013). Value shift also is a current topic of debate in conservation science (Manfredo et al. 2017; Ives and Fischer 2017). However, most of this discourse remains focused on interior change at the collective group (or societal) scale—that is, communities, and societies as a whole hold certain collective values which may or may not be conducive to sustainability. To date, scholars seem to have neglected the importance of individual inner lives, including their own. Yet, the inner lives of individuals have been (perhaps unsurprisingly) highlighted by those outside academic circles, especially in spiritual arenas. For example, Pope Francis in his Church Encyclical *Laudato Si* (On care for our common home) suggests “the ecological crisis is also a summons to profound interior conversion… I am interested in how such a spirituality can motivate us to a more passionate concern for the protection of our world” (Pope Francis 2015). Similarly, in “Ethics for the New Millennium”, the Dalai Lama (1999) argued that greater attention to our inner worlds would both lead to greater individual happiness, as well as provide a sound foundation for a more ethical and sustainable global community.

Against this background, our aims for this article are twofold: to highlight the neglect of our inner worlds in sustainability scholarship and practice, and to stimulate discussion of how engaging with our inner worlds may help effect change towards sustainability. We seek to speak as ‘mainstream’ sustainability scientists to other colleagues in our field, hoping to encourage members of our own field to begin to engage more deeply with the notion of inner worlds. In due course, this will necessarily entail bridging gaps to existing work from other disciplines, such as extensive scholarship on individuals’ inner worlds from branches of philosophy and psychology. Here, we do not try to complete this journey, but rather lay down arguments for why it will be worthwhile to start taking steps in that direction. To begin, we explore four realms of enquiry and how they have been emphasised in sustainability science over time.

## Viewing sustainability science through four realms of enquiry

Sustainability science has emerged as an integrative arena that brings together many disciplines with a focus on understanding the connections between human and natural systems so as to generate solutions for pressing planetary challenges. Sustainability science has been described as ‘use-inspired basic research’, highlighting its dual role of generating fundamental understandings of the world and providing practical solutions (Clark 2007). Yet, some domains of reality have been neglected in sustainability science. To understand this more fully, we distinguish between two dimensions of reality: an internally versus externally experienced dimension; and an individually versus collectively experienced dimension. Following Wilber (2000), we recognise that combining these two dimensions yields four domains of human experience, or four ways of generating knowledge about the world. These four dimensions can be labelled as follows: (1) ‘it’—knowledge of exterior and individual phenomena, (2) ‘they’—knowledge of exterior and collective phenomena and their interactions, (3) ‘we’—knowledge of internal and collective phenomena and their interactions, and (4) ‘I’—knowledge of internal and individual phenomena and experiences (Esbjörn-Hargens 2010). We show below how sustainability science relates to each of these four dimensions, and argue that the fourth dimension—‘I’—has been largely neglected to date. A summary of the four realms of enquiry is outlined in Table 1.

**Table 1:** Four dimensions of how humans understand and experience reality (c.f. Esbjörn-Hargens 2010), and their actual or potential contribution to sustainability science

Realm of enquiry	Mode of enquiry	Focus of enquiry	Insights for sustainability practice	Examples of sustainability questions
It	Empirical, positivist, reductionist	Biophysical	Composition of the exterior world (descriptive)	How much carbon is captured in permafrost?
They	Systems thinking, e.g. stocks, flows and feedbacks	Natural, social, or social-ecological systems, e.g. institutions and ecosystems	Dynamics of the exterior world, including change dynamics	What is the effect of climate change on permafrost, and which feedbacks result from permafrost melting?
We	Recognition of plurality, both qualitative and quantitative	Cultures	Recognising plurality in values to effect social and cultural change; increasing public participation	What are the implications of a post-truth culture in trying to address climate change?
I	Personal reflection and introspection	Personal experience and beliefs	Beliefs about what constitutes a ‘good life’; deep assumptions about what matters; mental wellbeing; psychological maturity; spiritual outlook	What is the inner basis for taking action to influence the exterior world? How can individuals tap into inner sources—e.g. spiritual, emotional, value-related—to resource and sustain creative (scientific and other) endeavour in the face of climate change in a post-truth culture?

### It: Exterior individual

The ‘it’ domain might be understood as empirical enquiry into the outside world. It focuses on understanding external phenomena, often in a quantitative way, and adopts an objectivist epistemology, which ensures the researcher is kept at a distance from the subject. Questions that are answered through this form of enquiry might relate to the chemical composition of a substance or its behaviour in different settings. This type of knowledge is sometimes connoted with the ‘pure sciences’, and has important contributions to make to sustainability. The ‘it’ quadrant is closely connected with ‘environmental science’, a precursive discipline to sustainability science. Topics of interest may include the amount of carbon stored in soil or the mineralogy of bedrock underlying a river basin.

### They: Exterior collective

This dimension is closely related to systems thinking. Sustainability science was established as a field that seeks to “understand the fundamental character of interactions between nature and society” (Kates et al. 2001, p. 641). In this way, a systems perspective has been central to the development of the field, focusing on relationships among system elements. These include the biotic and abiotic elements of ecosystems and the influence of social structures such as institutions and policies on these elements. Questions in this domain may include ‘what is the effect of the use of agricultural pesticides on river ecosystems?’ or ‘how do fishing quotas lead to recovery of fish populations?’. In this way, the ‘exterior-collective’ domain has been the primary focus of sustainability science to date. Major advances in sustainability science have been possible through employing systems thinking (Fischer et al. 2015).

### We: Interior collective

The “we” dimension describes collectively experienced, internal phenomena, such as social values. In recent years, sustainability scholars have begun to emphasise the importance of intangible and internal dimensions of human experience. Miller et al. (2014) for example, argued for the need to move beyond simply the analysis of sustainability problems to also consider social values. They state that “inquiries into values are largely absent from the mainstream sustainability science agenda. Yet, at its core, sustainability is a fundamentally ethical concept raising questions regarding the value of nature, responsibilities to future generations and social justice” (p. 241). This recognition of values has been framed in the context of collective groups, and has been tied closely with discourses of reflexive governance and participatory decision-making (Reed et al. 2010; Smith and Stirling 2017). The central argument has been that robust decisions for sustainability in a ‘post-normal’ world (Funtowicz and Ravetz 1994) require the careful integration of scientific knowledge with diverse and plural stakeholder values and perspectives (Colloff et al. 2017). The assessment of social values has therefore become a rapidly growing field of enquiry in sustainability and conservation (Ives and Kendal 2014; Kenter et al. 2015; Tadaki et al. 2017). Indeed, as Miller et al. (2014, p. 241) state “As soon as values become a core part of the sustainability research agenda, then the need for participatory approaches follows, since decisions can no longer be based solely on technical or scientific criteria (the domain of expert knowledge) alone”. Questions relevant to this domain include ‘what visions for sustainability do different stakeholders have?’ and ‘what sets of values are embedded in policy frameworks?’. Navigating a plurality of values, in turn, has major benefits for uncovering socially robust trajectories towards environmental sustainability (Kenter et al. 2015; Scholz and Steiner 2015).

### I: Interior individual

Finally, the “I” dimension relates to the inner worlds of individual people. Unlike the previous three domains, the interior-individual domain has been almost entirely neglected in sustainability science. The inner landscape of both sustainability scholars and members of communities that researchers investigate has been largely overlooked or seen as inaccessible. And yet, we argue that there is a fundamental relationship between our inner lives and the kind of sustainable future that we aspire to create. Science typically removes the subject of research from the investigator, but there is a need for greater integration. We concur with Wamsler et al. (2017) who call for “more sustainability research that acknowledges positive emotional connections, spirituality, and mindfulness in particular, recognizing that the micro and macro are mirrored and interrelated.” The interior lives of individuals might be understood as a ‘deep leverage point’ (Meadows 1999; Abson et al. 2017; Fischer and Riechers 2019) for change, because the goals, values, worldviews and emotions of people are the places from which the motivations and methods for pursuing sustainability originate and can be maintained. Key questions that this domain asks are ‘who?’ and ‘why?’. While other domains of investigation focus on the ‘what’ or ‘how’ of sustainability, this domain seeks to understand more deeply ‘who’ is pursuing sustainability, and ‘why’ an individual lives the way she does. Understanding our inner lives is central to this goal and a failure to look inwardly might compromise our ability to work effectively for (‘good’) change. Despite its lack of attention to inner worlds to date, given its position as an integrative arena, sustainability science may be ideally positioned to function as a boundary space to more fully capture these phenomena in the context of other dimensions of the world.

In talking about individuals’ inner worlds, we acknowledge that terminology is difficult and often ambiguous. We consider inner worlds to encapsulate entities of values, thoughts, emotions, identities, beliefs and worldviews, amongst others. As such, the term is broad and inclusive, so as to invite exchange of ideas and insights from across academic disciplines. We distinguish inner worlds from phenomena that exist in the ‘it’, ‘they’ and ‘we’ dimensions, which have been the primary focus of sustainability science to date. We recognise that the four domains we outline are a simplified abstraction for the purpose of aiding analysis: often it is in the connections between different domains that human experience of the world is understood. For example, many religious traditions engage interior dimensions via physical, embodied expressions of spirituality in community with other people. Indeed, Buber (1958) famously argued that human experience is summed up in interactions between individuals and objects (I-it relationships) and individuals and other people or the divine (I-thou relationships). Thus, while we discuss the four dimensions discretely, we consider it important to explore relations among these dimensions in the future.

## Inner worlds as a realm of transformation

**Fig. 1:**
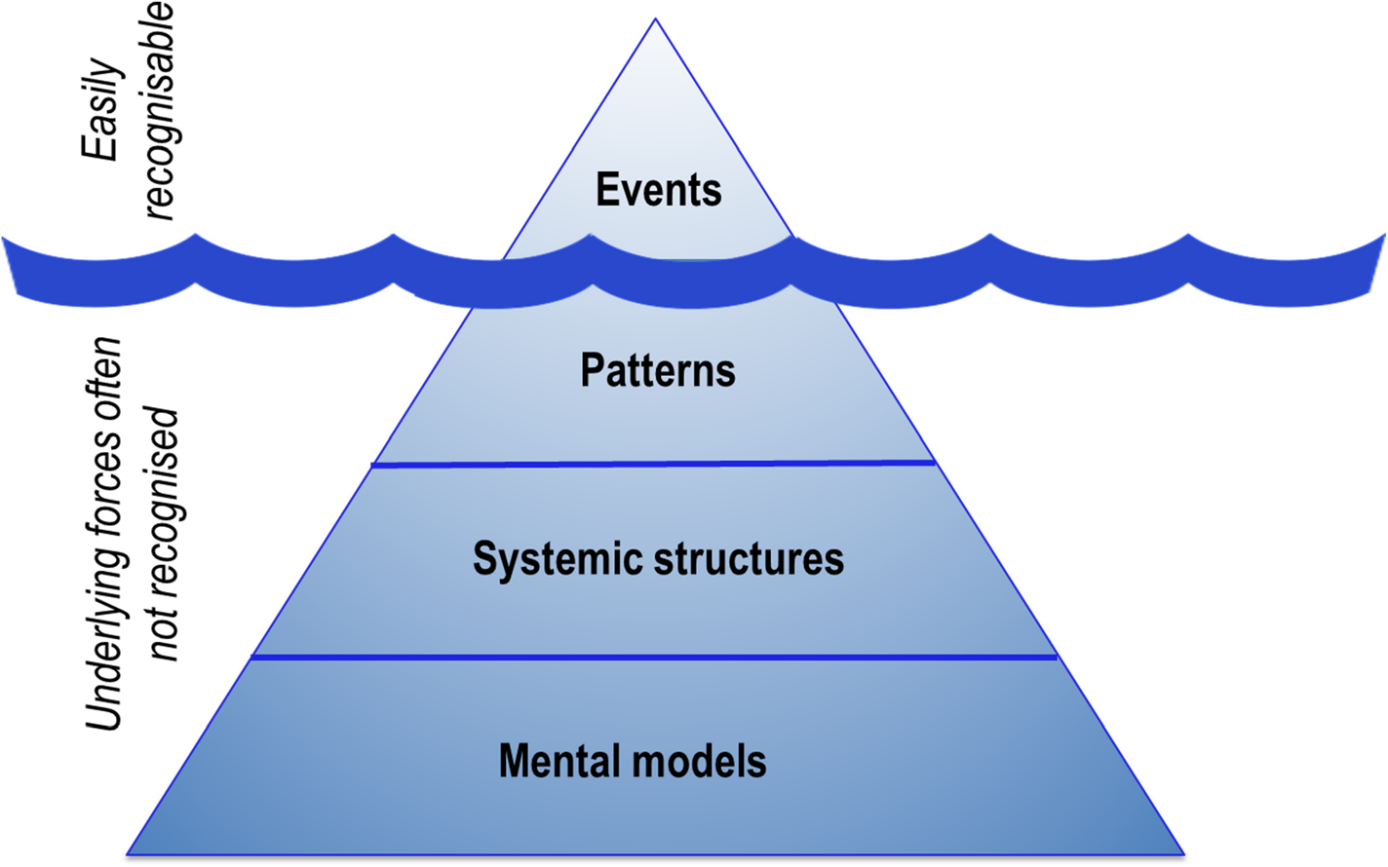
Four dimensions underpinning system function (adapted from WWF 2016; also see Nguyen and Bosch 2013)

Our inner worlds underpin much of how systems function, yet are commonly ‘beneath the surface’. One useful image to communicate this is by drawing on the analogy of an iceberg (Fig. 1). According to systems thinking, the deepest and most influential levels of a system are the underlying ‘mental models’: “the filters through which we interpret our experiences, evaluate plans and choose among possible courses of action” (Nguyen and Bosch 2013, p. 109). These are invisible but inform the questions we deem appropriate to ask, and underpin the structures, patterns and ultimately events that are observed and measured by scientific methods. The capacity for individuals to suspend assumptions, critique their mental models and potentially adopt new paradigms thus is one of the most powerful ways to dramatically influence sustainability outcomes (Meadows 1999).

We suggest that the sustainability crisis is in large part an emergent property of the state of our inner worlds. If we consider only external solutions to ‘out there’ problems (such as biodiversity loss, climate change, resource exploitation), we will fail to identify some of the most powerful and effective solutions that begin ‘in here’. It might be said that the scale of the sustainability crisis extends all the way from planetary systems to the heart and soul of every human being. In this way, we consider the inner life as both an underexplored *means* to change, and an *end* in itself. In short, since our inner lives underpin external change, we argue that change in the world must occur (in part) from the inside-out. Yet change must also occur from the outside-in: our inner lives must be shaped by the reality of the social and environmental injustices that are occurring in the world today. In this way, taking our inner lives seriously does not mean separating ourselves from external reality as a form of escapism. Rather, we argue for inner lives that reflect more closely the challenges of sustainability that are before us.

### The inner life as a means to sustainability outcomes

There are signs of an opening up of scientific horizons in sustainability science that could accommodate such an appreciation of inner lives. For example, effective action for sustainability is increasingly understood to require not only *systems knowledge* (technical knowledge of how systems function) but also *normative knowledge* (how systems ought to be), and *transformative knowledge* (how to change systems to more desirable states) (ProClim—Forum for Climate and Global Change 1997; Abson et al. 2014). The call for transformative science is premised on a commitment to not only study processes of transformation but to activate them, which necessarily involves shifts in the mindsets of many individual stakeholders, including sustainability scientists themselves (Schneidewind et al. 2016). The strongest step in this direction thus far is in sustainability science education and teaching (Caniglia et al. 2016; Wiek et al. 2016). The Aristotlean concept of *Phronesis* (practical wisdom) has also been recognised as essential for sustainability transformations (Fazey et al. 2018). We support these recent efforts to expand thinking in sustainability science and suggest that a focus on ‘inner worlds’ could help to create coherence in this emerging area of thought.

How can our inner lives influence sustainability? One vital area is through the motivational resources that exist in our inner lives. This includes deep awareness, building of empathy, and willingness to transcend paradigms. Awareness of our deepest motivations and experiences is perhaps the most fundamental (and grossly neglected) aspect of our inner worlds. Practices of individual reflection reveal awareness of society’s values and goals, our own values and goals, and differences between the two. Reflection can also help build empathy and compassion towards others by seeing matters from others’ points of view. Contemplation can even enable an expansion of empathy to include people from different cultures and locations, and non-human subjects (wildlife, ecosystems), which has been found to relate to pro-environmental behaviour (Berenguer 2003). This ‘shifting perspectives’ is a fundamental skill in enabling personal paradigms and mental models to be transcended. It is the malleability of personal paradigms that is the most powerful tool for transformative change (Meadows 1999; O’Brien 2018).

Inner life, with its values, goals and (often subconscious) desires, can be understood as the deepest driver of behaviour and behavioural change. Because sustainability ultimately requires behaviour shift (Schultz 2011), revealing, understanding (and potentially influencing) inner life is critical for developing strategies for change. Empathy cultivated via contemplation can be translated into action (Ericson et al. 2014). Paying attention to the inner life can ‘tap into’ something bigger than oneself. Such ‘transcendent’ motivation is common to all religious traditions, and has sustained action for profound social change throughout history. Nevertheless, while the inner life is a deep driver of behaviour, it is unlikely to be sufficient to generate the profound systemic change necessary for addressing global sustainability challenges in isolation. Any exploration of inner worlds within sustainability science must be done in conjunction with analysis of institutional structures, social context and politics (see O’Brien 2018).

### A healthy and compassionate inner life as a sustainability goal

Not only are our inner lives fundamental to the pursuit of social and environmental well-being, we suggest that the state of our inner lives ought also to be regarded as something worthwhile in its own right. In relation to the image of the iceberg, sustainability is greater than simply the events that occur (such as the use of renewable energy, or the provision of adequate housing). It necessarily includes the systems and structures that enable sustainability to be realised. A society free from violence thus cannot be called ‘sustainable’ if ‘peace’ is maintained through an oppressive dictatorship. In this way, sustainable actions and outcomes are not truly sustainable if motivated by greed or inner discord. At present, many sustainability strategies do not challenge the underlying values that contribute to it, but seek to work with these values (Manfredo et al. 2017). Tax incentives for ‘green’ products (e.g. electric vehicles) implicitly appeal to greed and materialism in order to shift behaviours. Similarly, sustainability scholars and activists can be driven by insecurity, fear or hubris just as much as other professionals. What if we extended to our own lives the aspiration of wellbeing and flourishing that we strive for in our sustainability work? Exploring inner lives, and working towards sustainability from the inside out, may reveal immaterial sources of lasting contentment and well-being, with positive flow-on effects for the world at large.

## How could inner life be approached in sustainability science?

Increasing recognition of the inner life in sustainability science is likely to be a long process. This article does not presume to provide a simple blueprint for how to address the neglect of the inner life. Yet, we offer below some starting points to a new pathway, which we hope will open conversation among sustainability scholars and practitioners. We consider that the concept of ‘leverage points’ for sustainability transformation (Abson et al. 2017; Fischer and Riechers 2019) is a useful framework by which this can be explored. According to Meadows (1999), complex systems possess different ‘leverage points’ whereby interventions can affect a certain amount of change. Shallow leverage points focus on existing system parameters. They are easily acted upon but unlikely to bring about transformative change. In contrast, deep leverage points tackle underlying worldviews, paradigms and values—they are more difficult to work with, but have much stronger transformative potential. We argue that a focus on the inner life has major potential to function as a domain for deep leverage for change. To operationalise this, we therefore call for (i) an expansion of the language used in framing sustainability, (ii) greater consideration of the inner life in sustainability research, and (iii) enhanced awareness and cultivation of the inner life in practice.

### Framing and language

The language used to articulate sustainability concepts and problems often betrays highly normative perspectives on the framing of sustainability. Lakoff and Johnson (1980) demonstrate that the language we use gives us clues to deep and collectively-held conceptual frameworks (and thus to the paradigms that shape them). We suspect that language contributes to a cycle, either virtuous or vicious: language expresses paradigms, and reinforces them. A change of language, in turn, has potential to challenge deeply held beliefs, and potentially shift them. Indeed, language might be considered a ‘deep leverage point’, acting to influence system paradigms. For instance, the term “sustainability science” implies a rational approach to the pursuit of maintenance. In contrast, other terms might connect with a deeper desire and inspire us to seek and create the futures we want. Rabinow (2011, p. 217) refers to a “flourishing” existence, supported by a science of “care”-ful “practices, relationships and experiences”. Stengers (1997, p. 113) writes about (re)awakening a “jouissance” in science, which has potential to bridge the gap between the “intensity” of scientific discovery, and the “sterilizing” language often used to express it. Wahl (2016) also promotes the concept of “regenerative cultures” over sustainability. Even use of the term “the environment” has recently been challenged within public discourse (Monbiot 2017).

Given the importance of language, we call for a greater exploration and expansion of terminology in sustainability that engages both the head and the heart. The term we introduced in this article—inner worlds—is deliberately broad and encompasses many dimensions of internal human phenomena; including, as we outlined above, emotions, thoughts, identities and beliefs. While traditional science typically strives for great conceptual precision, seeking to create sharp boundaries between related concepts (e.g. the distinctions between attitudes, beliefs and values in psychology; Rokeach 1968), such precision can at times constrain integrative enquiry and thereby obscure important insights. Scientific language has also not arisen to develop mindfulness and empathy. We offer the term “inner worlds” as a way of holding together multiple dimensions of “human being” that are otherwise neglected in sustainability science. Similarly to the term “resilience”, the vagueness of the term “inner worlds” thus could be considered an asset, in accordance with Strunz’s (2012) argument that a certain degree of conceptual vagueness fosters creativity and enables integration across different knowledge domains. The term “inner worlds” thus could help to bring together existing insights, and perhaps generate new ones, with tangible benefits for both sustainability research and practice.

### Research

There are a number of potential research questions salient to how our inner worlds connect with sustainability. We explore a few here, recognising that this list is nowhere near exhaustive. The first set of questions refers to how inner lives of individuals relate to individual behaviours towards sustainability. One dimension of the inner life that is particularly pertinent is that of values. While much has been written in social psychology on the relationship between personal values and behaviours (Dietz et al. 2005; Steg and Vlek 2009), the focus in the context of sustainability has been on values as they exist in a certain population or in a collective sense. For example, there is a voluminous literature on the structure and persistence of human values across different cultures and socio-political contexts (e.g. Schwartz 1994; Inglehart et al. 1998). In contrast, there has been little exploration of personal values as preconditions for action in support of transformative change for sustainability (Ives and Fischer 2017). The importance of personal values in the context of organisational leadership is one area where the relationship of personal dimensions to higher level systemic change is directly relevant (e.g. Hemingway and Maclagan 2004). Of course, values are only one facet of the inner life and should not necessarily be separated from other dimensions of inner experience. There is also a need to explore how other conditions of people’s ‘inner’ lives (such as emotional wellbeing, or capacity for reflection) can enable and motivate actions for sustainability. One area of promising research is the relationship between personal character strengths and virtues and sustainable behaviour (Corral-Verdugo et al. 2015).

The second field of research is how inner worlds can be shaped and transformed to align more with sustainability outcomes. The capacity for personal values to be shaped and shifted intentionally is gathering greater interest (see Raymond and Kenter 2016), and there is a need to explore how such value shift might enable sustainability transformation (Ives and Fischer 2017). The fostering of ‘virtues’ is another growing field of study that relates deeply to sustainability. Traditional western virtues include humility, kindness, patience, diligence, temperance and charity. Individuals who have inner lives characterised by these qualities may, arguably, be positioned to pursue sustainability passionately and persistently. The need to emphasise virtues in education is increasingly recognised, as the inadequacy of knowledge and skills alone in contributing to a healthy and flourishing society is acknowledged (see Arthur et al. 2017). How such virtues might be cultivated within individuals and how they relate to change for sustainability is therefore an arena ripe for further research.

A third arena for further research is how institutions and organisations that relate to the inner life might promote sustainability. This includes religious groups and communities, and their institutionalised practices such as mindfulness, meditation and contemplation. With 84% of the global population professing some kind of religious faith (Pew Research Centre 2017), religious institutions are ideally positioned to engage with the inner lives of individuals as they relate to sustainability and to promote inner change. There is therefore a need for research into how various spiritual and religious beliefs and practices might motivate or constrain action for sustainability (Hitzhusen and Tucker 2013). While research has shown somewhat complicated relationships between religiosity and pro-environmental behaviour (Gifford and Nilsson 2014), there is undoubtedly a need to engage spirituality with the sustainability crisis, and religious institutions are ideally situated to do this. As Orr (2002) noted “The transition to sustainability will require learning how to recognize and resolve divergent problems, which is to say a higher level of spiritual awareness”.

Finally, there is a need for research on how inner worlds relate to existing theories of social change. Many theories have been proposed, investigated and operationalised. These do not need to be superseded by a ‘new’ theory of change focused on inner worlds, but rather, understanding inner worlds and their relationship to other quadrants (outlined in Sect. 2) opens up a broader perspective from which new questions can emerge. We have already introduced the concept of ‘leverage points’ as a theory of change grounded in systems thinking (Abson et al. 2017). In this context we see inner worlds as sources of leverage as they can connect observation to realisation and action. They enable dynamics in the other quadrants to be seen and their significance felt, including dynamics of power (the interior-collective dimension ‘we’), systems of injustice and unsustainability (the exterior-collective quadrant ‘they’), and changes in the biophysical world (the exterior-collective quadrant ‘it’). Actively incorporating inner worlds into our analyses would mitigate against the risk of divorcing interior and individual catalysts for change from the larger set of contexts deserving of change. Similar conceptual and empirical research should be done to relate inner worlds to other theories of social-ecological change. Below are a few examples. First, psychologically-grounded causal theories of behaviour, such as Ajzen’s Theory of Planned Behaviour (Ajzen 1991) and Stern’s Value, Belief, Norm Theory (Stern and Dietz 1994) continue to dominate literature on behaviour-change policy. Consideration of inner worlds could inform how deeply held values are formed and shaped over time, and in response to human interaction and various contexts. Second, social practice theory emphasises the importance of routines and behaviours within distinct social contexts (Shove 2010). There is an opportunity to explore how such practices stem from and influence individuals’ inner lives. Finally, social innovation theory explores the emergence of new social solutions to problems within various institutions (Moore and Westley 2011). Considering inner worlds could highlight the inner ‘preconditions’ for innovation and the meanings of these innovations as they emerge.

### Practice

Of equal importance to undertaking research on the inner life and its relevance to sustainability is the fostering of healthy inner lives of sustainability professionals. In essence, there is a need to ‘lower the water line’ of the iceberg (Fig. 1)—to increasingly expose those invisible dimensions (such as mental models and emotions) that influence the external activities and events we pursue. Structural change in academic institutions may be necessary to combat the increasingly competitive, output-driven and performance-oriented cultures in many universities (Fischer et al. 2012a) to help promote inner health and well-being of faculty staff. Practically, this may entail providing opportunities (both places and times) for reflection and informal exchange with colleagues (Fischer et al. 2012b), promotion of training and development in inner virtues and inner transitions (both for faculty and students), and prioritising aesthetics and meaning in work. Such a shift is may be enabled and reinforced by modifying existing systems and processes. These could include criteria for academic honours and promotion incorporating elements of personal character strengths, or funding bodies looking beyond criteria related to academic output and external ‘impact’ to also reward sensitive, respectful modes of working and provide resources for cultivation of inner health and well-being. Innovative teaching programmes are likely to be an important part of a sector-wide shift towards appreciation of inner worlds, both within traditional institutions (e.g. teaching on sustainability and inner transformation at Lund University, or the role of inner worlds in environmental leadership at the University of Nottingham), as well as pioneering educational platforms (e.g. Ubiquity University’s Wisdom School).

Personal practices are also likely to be important in embracing inner worlds in sustainability. In the context of a “post-truth” society that is increasingly skeptical or dismissive of scientific evidence, there is a need for sustainability scholars and practitioners to take time to create space to build the inner resources that will sustain action over the long term. Practices of solitude and silence have long been held as vital to inner health and wellbeing amongst many religious traditions. Mindfulness techniques have been shown to reduce stress and promote mental health (Grossman et al. 2004), and the potential for these to contribute to sustainability has been recognised recently (Wamsler et al. 2017; Wamsler 2018). We are interested in how participation in these practices could help bring together the inner reality of our lives with the kind of world that sustainability scientists aspire to see.

## Conclusion

The persistent degradation of the biosphere despite growing scientific knowledge suggests that there is a need for sustainability science to take a look at some of the deeper drivers of anthropogenic planetary change. We have argued that sustainability science has neglected an important dimension of human experience—the inner worlds of individuals. These have the potential to fundamentally shape human behaviour and possibly even the functioning of social systems. We call for greater recognition of the inner life in sustainability science and for a new agenda of research and practice that highlights the inner revolution that is needed. With a greater awareness and activation of inner resources for sustainability, we might just locate the transformative capacity to bring about the change necessary for a safe, just and sustainable future for humanity and the planet.
